# Developing Insulating Polymeric Foams: Strategies and Research Needs from a Circular Economy Perspective

**DOI:** 10.3390/ma15186212

**Published:** 2022-09-07

**Authors:** Lucia Doyle, Ingo Weidlich, Ernesto Di Maio

**Affiliations:** 1 Technical Infrastructure Management, HafenCity University, 20457 Hamburg, Germany; 2 Dipartimento di Ingegneria Chimica, dei Materiali e della Produzione Industriale, University of Naples Federico II, 80138 Naples, Italy

**Keywords:** insulation, polymer, foaming, circular economy, cradle2cradle, green engineering, circular design

## Abstract

Insulating polymeric foams have an important role to play in increasing energy efficiency and therefore contributing to combating climate change. Their development in recent years has been driven towards the reduction of thermal conductivity and achievement of the required mechanical properties as main targets towards sustainability. This perception of sustainability has overseen the choice of raw materials, which are often toxic, or has placed research efforts on optimizing one constituent while the other necessary reactants remain hazardous. The transition to the circular economy requires a holistic understanding of sustainability and a shift in design methodology and the resulting research focus. This paper identifies research needs and possible strategies for polymeric foam development compatible with Circular Product Design and Green Engineering, based on an extensive literature review. Identified research needs include material characterization of a broader spectrum of polymer melt–gas solutions, ageing behavior, tailoring of the polymer chains, detailed understanding and modeling of the effects of shear on cell nucleation, and the upscaling of processing tools allowing for high and defined pressure drop rates.

## 1. Introduction

Insulation materials are covered by the European Eco-design Directive [1]. Eco-design, from the design process standpoint, can be referred to as a relative approach. It “starts with the present state of affairs and identifies existing problems, which people subsequently attempt to solve” [2]. Although bringing improvement, this approach has been criticized for limiting the search for truly sustainable innovations, as it only proposes optimizations from what is existing [3]. The focus of this relative approach is ‘not the good, but the less bad’ [4]. Eco-design strives for a wide implementation of insulation materials, seeking energy efficiency. Under the assumption that ‘the major environmental impacts of insulation lie in the environmental benefits it provides during the use phase’ [5], research has been driven by the objective of reducing thermal conductivity and matching the required mechanical properties as the only viewpoint, assuming that this is sustainable as such.

This has led, for example, to the selection of chlorofluorocarbon (CFC) first and flammable hydrocarbons (i.e., pentane) at present as blowing agents [6], as they present lower thermal conductivity than other inert gases, while at the same time increasing the need for flame retardants, which are often toxic and hinder the later recycling of the material [7]. Another paradoxical example could be the research efforts placed on green polyurethane foams by deriving polyols from vegetable oils, such as palm [8,9,10], rapeseed [11], soybean [12,13] and linseed [14,15], while diisocyanates are still required for the polyurethanes’ synthesis, which are classified as suspected of causing cancer, as dermal and respiratory sensitizers, for acute toxicity following inhalation, as well as eye, skin and respiratory irritants [16] and have recently been restricted [17].

The Circular Economy (CE) is gaining momentum. Increasing focus on the development of circular buildings [18,19,20] and infrastructure [21,22,23,24] requires the availability of circular materials and products. The availability of circular, sustainable and recyclable insulation materials is reported to be particularly lacking [18], which shows the need for research and development in this direction. The fundamental distinctions between circular product design versus eco-design have been the focus of methodological research [3]. Circular Product Design requires a holistic approach to ‘closing the loop’ of product life cycles by extracting the maximum value of all raw materials and widespread recycling and reuse [25]. However, recycling and reuse can be hampered by the presence of certain chemicals because of technical barriers or because of their hazardous nature. Moving toward the CE will require a paradigm shift in the way things are produced, i.e., using less additives and eliminating toxic chemicals to enable mainstream recycling.

This paper takes the Cradle-to-Cradle framework [4] and will use the 12 Principles of Green Engineering [26] and the Inertia Principle [27] as guidelines to measure the fulfillment of Circular Design, as detailed in Section 2. Although sustainability and environmental concerns have supported different research lines in polymeric foaming, such as the elimination of CFCs [28,29,30,31], the use of inert gases as blowing agents [32,33,34,35,36], the foaming of thermoplastic materials [32,37,38,39], biobased [40,41,42] or biodegradable [33,43,44], and this typically involves only one aspect of the foam developed, and none of these works considers or consistently applies the principles of Green Engineering or Cradle to Cradle.

Reviews of the literature on recent trends in polymer foaming, including knowledge gaps, have been published in recent years [45,46,47]. These works provide a general overview of the state of the art and current technical challenges. The novelty of this work is that it presents a broad screening of the literature through a Circular Design filter, to answer the following questions: how can polymeric foams be developed while consistently applying the Cradle to Cradle framework? Which research gaps need to be addressed? The recycling possibilities of traditional polymeric foams is out of the scope of this paper.

## 2. Designing for the Circular Economy

As to develop an insulating foam that meets the requirements of the circular economy, the definition and criteria for this fulfillment need to be established.

The concept of CE is fed by different schools of thought, and a description of them can be found in [48]. This section summarizes the essential concepts and applicability of the selected frameworks and principles, which are Cradle to Cradle [4], the 12 Principles of Green Engineering [26] and The Inertia Principle [27]. In this paper, the term Cradle to Cradle design and circular product design will be used indistinctly. It should be stressed that Cradle to Cradle is here referred to as the design methodology, and not to the scope or boundaries of a Life Cycle Assessment (LCA). LCA is a tool for the assessment of environmental impacts associated with established products or processes, while this paper and the Cradle to Cradle design methodology is concerned with the design and development of products and processes. Discussions on the similarities and differences can be found in [49].

### 2.1. The Performance Economy and the Inertia Principle

With the objective of decoupling growth from resource consumption, the Performance Economy [27] proposes business models based on trading performance instead of goods. Through such business models, the internalization of waste costs is achieved and resource efficiency is rewarded. Walter Stahler introduced a guiding principle for circular design with the Inertia Principle: “Do not repair what is not broken, do not remanufacture something that can be repaired, do not recycle a product that can be remanufactured. Replace or treat only the smallest possible part to maintain the existing economic value of the technical system” [27]. The aim of the Inertia Principle is to maintain the integrity of the product for as long as possible, minimizing the environmental costs of the required processes to restore the economic value of the product. Transferring the concept to polymeric foams would prioritize thermoplastic foams over chemically recyclable foams, for example, as a lower level of recycling is needed.

### 2.2. Cradle-to-Cradle Design

The cradle-to-cradle (C2C) design [4] antagonizes current recycling in that it starts from the beginning (design for recycling), rather than the current end-of-pipe approach (what to do with waste). It proposes a new approach towards sustainable design based on the intelligence of natural systems.

Three main tenets are proposed:

- Waste equals food;
- Use of current solar income;
- Celebrate diversity.

The first tenet brings the notion that waste is a human based concept, and by replicating nature, materials should be designed as nutrients that flow through the biological or technical cycle.

In the biological cycle, biobased materials would be used and composted after their lifecycle, serving as nutrients for crops to grow and allow the circular production of new biobased materials. The technical cycle is a closed-loop system in which non-harmful, valuable synthetic and mineral materials flow in manufacturing, use, recovery, and remanufacture cycles. This definition implies no preference for bio or non-bio materials, as long as they are fully recyclable and non-harmful. An important issue is to keep separate the biological and technical cycles, to enable the correct flow of materials. Hybrid bio/technical materials which cannot be later separated impeach their later recovery, as they cannot be composted nor technically recycled. This provides a first design criterion. Material safety plays a central role and is a fundamental criterion in the design. The second tenet sets renewable energy as a source to power any C2C product or process, as nature uses photosynthesis. Thus, by keeping materials in the circular economy and powering cycles with renewable energy, the number of cycles a product or process is subjected to would be irrelevant from the environmental impact perspective. However, the fact that today 100% renewable energy is not yet available to all, and the choice of energy source supply for i.e., manufacturing facility is not always in the scope of the engineer’s work is a source of criticism towards the broad applicability of the framework [50]. The third tenet encourages to tailor designs to maximize their positive effects on the particular niche in which they will be implemented, or in McDonough and Braungart’s words, ecoeffectivity vs. ecoefficiency.

### 2.3. The 12 Principles of Green Engineering

The 12 principles of Green Engineering [26] provide a guideline for scientists and engineers aiming to design new materials, products, processes, and systems that are benign to human health and the environment. The principles are envisaged for broad applicability, from the construction of chemical compounds to urban architecture. Some examples include ‘designers need to strive to ensure that all material and energy inputs and outputs are as inherently nonhazardous as possible’ (Principle 1), ‘system components should be output pulled rather than input pushed through the use of energy and materials’ (Principle 5), and ‘multicomponent products should strive for material unification to promote disassembly and value retention’ (Principle 9). In the context of polymer foaming, the application of Principle 1 would support the need to phase out polyurethane due to the toxicity of diisocyanates.

The application of Principle 5 can be exemplified using shear forces to promote cell nucleation, where the presence of high shear would “pull” the gas phase out of the solid cavity [51], as will be discussed in Section 4.3. Principle 9 aims to minimize the use of additives. For the full list of principles and their details, the reader is referred to Anastas and Zimmerman [26].

As stated by the authors of both frameworks [52], the C2C vision sets the course for ‘what do I do?’ while the 12 Principles of Green Engineering answer “How do I do it?” In this sense, they will be used to define the research needs for developing insulating foams for the circular economy.

## 3. Methods

The objective of this paper is to identify the research needs and potential strategies for polymeric foam development from a circular economy perspective, where the meaning of a circular economy perspective is fulfilling the criteria described in Section 2. Data were retrieved through a literature search. As search engines, Google Scholar, Google Patents, and Espacenet were used. Used key words included foaming, polymeric foam, foamable, AND recyclable, recycled, thermoplastic, environmentally friendly, sustainable, high temperature, thermally stable, and insulation. Screened documents included peer reviewed journal articles, books and handbooks, dissertations, project reports, and patents. Relevant references identified in the evaluated pieces of literature were additionally included for completeness.

The retrieved literature was screened and the data were sorted to answer the following questions:

- What characteristics does a polymer need to have for successful foaming?
- Which are the main morphology–properties relationships of cellular plastics and the link to processing conditions?
- Which strategies can be followed to use these relationships and what research needs to arise when developing insulating polymeric foams according to circular product design?

Data collection, screening, and sorting was conducted in the period 2019–2022.

## 4. Results

The properties of polymeric foams are related to the properties of the polymer matrix and to the foam morphology, which in turn is connected to the relative density and the geometric structure of the foam [53]. At the same time, the final foam morphology itself is conditioned by the selection of polymer, blowing agent, and expansion techniques [6,54]. Developing a foam with target properties involves the selection of raw materials, process conditions, and foaming tooling and technology. The following sections evaluate each of these aspects and the research needs arising from them when following Circular Product Design.

### 4.1. Selection of Raw Materials: Need for Material Characterization

Material selection plays a central role, as it defines the properties of the final product and at the same time their nonhazardous and recyclable nature are at the heart of C2C design. The fulfillment of the full recyclability criteria implies no preference between bio-based or non-bio-based materials. A recent review comparing LCAs of fossil based and bio-based polymers found it was not possible to conclusively declare any polymer type as having the least environmental impact in any category [55]. Therefore, they will not be discussed separately in this paper. While the use of different additives is common in foaming, such as cell stabilizers [56,57,58], flame retardants [56,58,59], nucleating agents [32,58,59], and fillers [60,61], material diversity should be minimized to facilitate the later recycling of the product.

In line with the Inertia Principle [27], thermoplastics would be preferred to thermosets, as they retain greater product integrity, followed by chemically recyclable polymers. In line with the 12 principles of Green Engineering [26], the non-hazardous nature of the materials should be placed first. The requirement to replace commonly used foams because of their non-recyclability or hazardous nature of raw materials triggers the need for alternative foaming polymers. Insulating foams are often part of multi-functional sandwich structures. The application of circular product design to the complete structure may trigger the interest to foam particular polymers to match the other material layers [62]. However, the foamability performance assessment is not trivial. When comparing resin properties, PET can be expected to provide superior mechanical and thermal performance as well as improved chemical and flame resistance than PS [63], leading to a reduction of additives requirement. However, PET has long been a challenge in foaming, related to its low melt strength [54,64,65]. Likewise, the IR-absorbing characteristics of PLA arising from the ester group anticipate it to have better insulating performance than PS foam [36], but its foaming has again been found to be problematic, due to its low melt strength [40]. Polybutene-1 s high heat deflection temperature [66] and low thermal conductivity of 0.114 W/mK [67] make it a great candidate for insulation where heat resistance is required, such as district heating pipes. However, its foamability is only recently being explored [62].

**Figure 1:**
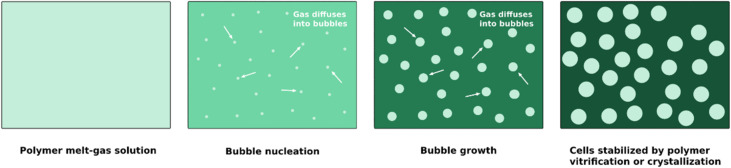
Cell nucleation, growth, and stabilization.

The basic principles of foaming can be found in the literature [6,35,45,68] and involve the sorption of the blowing agent into the polymer matrix under pressure, and the nucleation and growth of bubbles, which can be induced through a reduction in pressure or an increase in temperature. There is mass transport from the blowing agent in the polymer–gas solution to the bubbles, which ends with the vitrification or crystallization of the polymer. This last stage is critical for the success of the foaming process, as it stabilizes the cellular structure. A schematic illustration of the process is presented in Figure 1. Delayed or slow vitrification or crystallization may result in a too large extensional elongation of the cell walls, resulting in cell wall rupture and coalescence.

**Figure 2:**
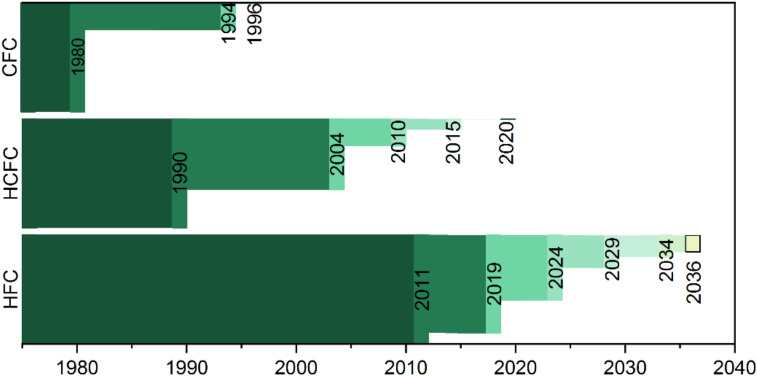
Sankey diagram representing blowing agents phase out for non-article 5 countries (industrialized) agreed in the Montreal Protocol and Kigali amendment.

Blowing agents are a necessary constituent of polymeric foams. They may be divided into chemical (CBA) or physical blowing agents (PBA). Physical blowing agents are generally preferred for lower density foams, while CBAs are preferred for high- and medium-density foams [6,64]. Within PBA, chlorofluorocarbons (CFC), hydrochlorofluorocarbons (HCFC), hydrofluorocarbons (HFC), hydrocarbons (HC), and inert gases (N_2_, CO_2_) have been used [6]. Before the mid-1980s, CFCs were preferred due to their soluble, volatile, and nontoxic nature [6]. However, their ozone depleting nature is well known, and the Montreal Protocol signed in 1987 [69] called for the phase out of their manufacturing and use, initially causing a shift to transition replacement HCFCs, and later to HFCs. These are now called to phase out due to their high global warming potential (GWP) by the Kigali Amendment to the Montreal Protocol [70] in force from 1 January 2019.The phase-out program is represented in Figure 2.

Today, hydrocarbons, despite flammable in nature, are the blowing agent of choice for extrusion foaming of insulating foams [6]. This flammable nature is a hazard [71,72], and typically requires further adding flame retardants, which are often toxic themselves [7]. Therefore, focus should be placed on the use of inert gases. There is currently increased interest in the use of CO_2_ as a blowing agent due to the mentioned safety and environmental reasons, as well as cost considerations [36,43,73,74]. The main drawback of its use is its higher diffusivity than that of long-chain hydrocarbons such as pentane, which affects the maximum expansion ratio achievable [32], the thermal aging of the foam [75], and can cause post-foaming shrinkage [72,76,77]. Strategies to overcome this will be discussed in Section 4.2.2. An alternative is the use of hydrofluoroolefines (HFO), labeled 4th generation blowing agents or refrigerants [78,79]. The main advantage of these blowing agents is their very low global warming potential [79,80,81], ozone depletion potential [79,81], and flammability [79], while presenting thermal conductivity similar to that of HFCs [82]. They also have a low photochemical ozone creation potential [80]. This has motivated their proposal [81,83] and evaluation [78,80,84] as blowing agents for polymer foaming, including PU [78,81,83], HDPE [81,83], PBT [81], phenolic resins [81], PP [81,83], PS [81,83], PTFE [81], PVC [81], PET [83] and cellulose acetate [84]. However, the toxicity of HFO is conflictive and depends on the particular HFO in question [80,85,86]. Some are reported to be toxic, such as HFO-1225 [85], while limited information prevents a conclusive statement for others, such as HFO-1234yf, which undergoes atmospheric degradation producing trifluoroacetic acid (TFA) [86]. There is reportedly no clear evidence of any HFO molecule being toxicologically innocuous [85]. Thus, further research on the use of HFOs as blowing agents for polymer foaming cannot be recommended.

The changes experienced and overcome by the foaming industry in the past 30 years demonstrate how a shift towards a more sustainable design and manufacturing of polymeric foams is possible when the right framework and R&D investments are in place. Since the number of inert gases is small and known, as for research needs concerning PBA, inert gases solubility and diffusivity in different polymer matrixes, the rheology of the polymer melt–gas solution and processing parameters can be cited and will be further discussed in the following section.

Chemical blowing agents are solid or liquid materials which decompose under certain conditions generating vapors [87]. An overview of the different blowing agents and their mechanisms of action can be found in [81]. In terms of Circular Economy, the innocuousness of the agent as well as of its decomposition products is a main selection criterion. Some of the solid residues from the decomposition of CBAs such as 5-Phenyltetrazol, azodicarbonamide or hydrazides are hazardous [88] and hence make these compounds ineligible. The CBA’s masterbatch carrier should be compatible with the polymer matrix. Since it is typically impossible to use the same polymer as the matrix and master batch carrier, to eliminate the risk of decomposition during production [88], using CBA’s involves polymer blending to some extent, and its impact on later recycling options, should be explored.

**Figure 3:**
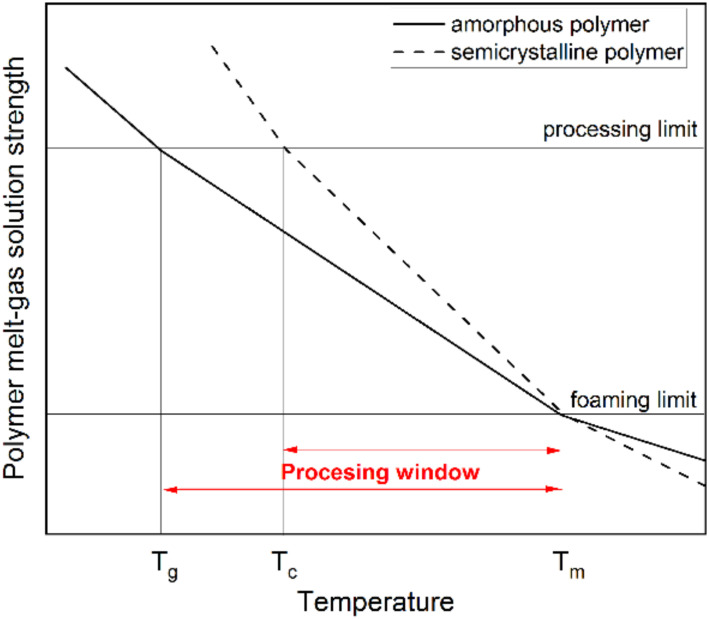
Schematic representation of the foaming processing window for semicrystalline and amorphous polymers, adapted from [6].

Concerning the polymer, the foamability of polymers is strongly related to their rheological behavior [74,89,90]. Characteristics commonly reported as required for successful foaming include high melt strength to withstand the elongational stresses acting during the bubble growth phase [32,33,40,64,65,91,92] and strain hardening as an enhancement of melt strength, contributing to the success of cell stabilization [43,89,90,93,94] and widening the processing window [90]. Strain hardening behavior would contribute to successful foaming when it occurs at the strain rates relevant to the foaming process [95], which are reported to be between 1 and 5 s^−1^ [74], although it has been suggested that the quantitative increase in the achieved expansion ratio might not remain when the process is scaled-up from laboratory to production scale [96]. Other important aspects include the size of the processing window, which is found between the melting temperature (T_m_) and the crystallization temperature (T_c_) for semicrystalline polymers or the glass transition temperature (T_g_) for the case of amorphous polymers [45,97]. The processing window is schematically represented in Figure 3. It has recently been reported that the crystallization rate and processing window could be of higher importance towards the consecution of low-density foams with fine morphology [98].

However, data on the extensional rheology of polymer melts are not readily available for all polymers [74].

Basic research is needed in polymer rheology characterization, to increase knowledge and widen the choice of foamable polymers. However, while the rheology of the polymer melt provides good starting information, the foaming process is determined by the rheology of the polymer melt–gas solution [74,99]. It is well known that the dissolution of the blowing agent can cause a plasticization effect on the polymer matrix [43,97,100,101], which results in depressing the T_g_ and T_m_. During bubble growth, a rapid viscosity increase can occur as the gas dissolved in the melt diffuses into the cells if the processing temperature is in the vicinity of the glass transition temperature of an amorphous polymer [43,100]. In semicrystalline polymers, this is more complex, as the increase in free volume has an impact not only on the crystallization temperature but also on its kinetics [43]. The crystallization kinetics and T_c_ have been established as relevant factors for the foamability of semi-crystalline polymers [43], and the knowledge of crystallization kinetics under the presence of blowing agent is pointed out as a knowledge gap to overcome for successful foaming optimization of each polymer–gas system under study [36]. It has significant effects both on cell nucleation mechanisms and cell growth [43,102], as well as on the cell stabilization step [36]. The semi-crystalline nature involves a sudden transition from a low viscosity material above the crystalline melting temperature (T_c_) to a high viscosity polymer below the T_c_ [63]. This leads to a narrower processing window than for amorphous polymers.

Moreover, the gas does not dissolve in the crystals, resulting in lower gas solubility [36,43,102,103].

**Figure 4:**
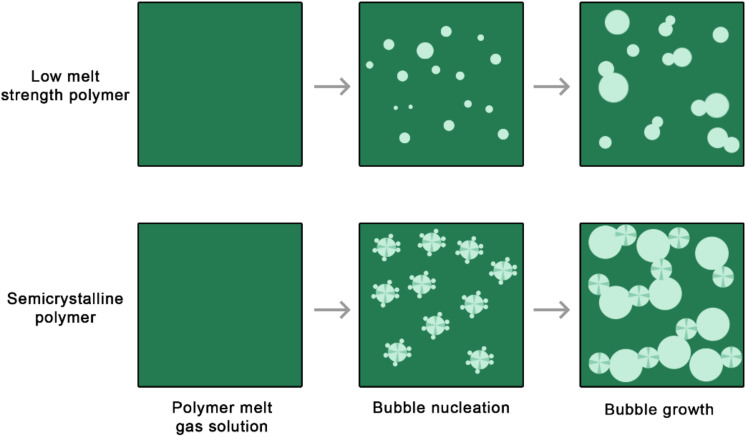
Low melt strength polymers do not yield foams with suitable morphology due to cell coalescence (**top** scheme). Crystal formation during the foaming of semicrystalline polymers can restrict cell coalescence (**bottom** scheme).

However, crystallization can also improve the foaming of low melt strength polymers by restricting cell coalescence [36], as schematically represented in Figure 4. An optimal crystallization degree during processing is needed to ensure that the foam has a high expansion ratio and a considerable cell density.

Lastly, the solubility and diffusivity of the gas in the melt [104] and the interfacial tension [45] condition the nucleation of the bubbles and the growth rate, hence the morphology of the foam [43,54]. The surface tension of a polymer–gas solution is lower than that of the pure polymer and intimately related to the gas concentration and polymer–gas system [33]. These data are not commonly available. For the case of extrusion foaming, the solubility limit at the given processing conditions marks the maximum mass of the blowing agent that can be added, to ensure a complete mixing and dissolution into the polymer, which would otherwise lead to premature bubble formation and inhomogeneous foam morphology [105]. This highlights the importance of determining the solubility of the blowing agent in the polymer at different pressures and temperatures.

Looking into the foam’s application, the characterization of the ageing of the foam is vital to confirm the fulfilment of the required properties during the required service life, which for insulation products spans decades. This reaches a further dimension from the circular economy perspective, as the different product lifecycle stages should be considered. While crystallinity challenges the foam processing, it could have positive consequences concerning the aging of the foam, since the crystalline regions create a more restrictive pathway for the diffusing molecules, hence decreasing the diffusivity of the blowing agent [102] out of the cells. Together with the permeability, it can play a major role on the ageing of the thermal properties of the insulating foam. The thermal conductivity of a foam (λ*_foam_*) is the sum of the thermal conductivity due to conduction in the polymer matrix (λ*_pol_*), the thermal conductivity due to conduction in the gas (λ*_gas_*) and the thermal conductivity due to radiation (λ*_rad_*) [106,107]:

λ*_foam_*(*t*) = λ*_gas_*(*t*) + λ*_pol_* + λ*_rad_* (1)

The contribution of convection is typically disregarded as it is reported to only occur when the cell size exceeds a few millimeters, with references stating the threshold at 3 mm [108], 4 mm [109] or 5 mm [110], the temperature is low, or the temperature gradient is very high [111]. Since the composition of the cell gas changes over time as the blowing agent diffuses out of the foam and ambient air diffuses in, so does the thermal conductivity of the foam. For reference, λ_cyclopenthane_ = 0.0110 W/mK, λ_CO_2__ = 0.0165 W/mK and λ_N_2__ = 0.0258 W/mK, as compiled in [87].

**Figure 5:**
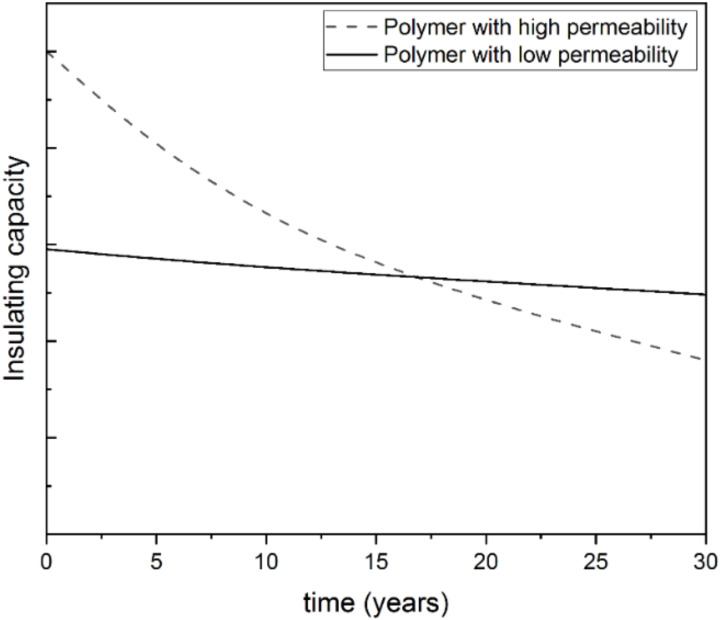
Conceptual representation of the long-term insulating capacity of foams depending on the permeability of the polymer.

Mangs [106] studied the ageing of the insulating capacity of PET and PU foams and determined the effective diffusion coefficients of CO_2_, N_2_ and O_2_ in PET and PU foams, and concluded they are 10 to 30 times lower in the PET foam, in the temperature range studied from 23 to 90 °C. This leads to a 10-times slower decrease in insulation capacity for PET than for PU foam [106]. Consistent with this, Ref. [111] reported effective diffusion coefficients of oxygen, nitrogen, and carbon dioxide in PET foams approximately 5–15 times lower than those in PU foam. With these data, a typical district heating pipe insulated with PU and a 3 mm polyethylene casing of dimensions DN 40/125 would decrease its insulation capacity by 16% after 30 years, while a pipe with PET foam with the same dimensions and a 1 mm PET casing would see a decrease of 3% [111]. Therefore, while the initial thermal conductivity of PET foam is higher than that of PU, associated with a larger cell size [106,111], the difference may be offset over time, as conceptually illustrated in Figure 5.

Crystallinity can also be used to modify the mechanical properties and heat resistance of the produced foams. There is a strong relation between heat resistance and crystallinity of a material, since the crystalline regions could sustain the material stiffness past its T_g_ [112]. Modifications in crystallinity could be introduced during resin manufacturing. However, the foaming process itself can enhance crystallinity, due to the free volume allowed by the dissolved blowing agent and the biaxial stretching during bubble growth. This is reported to allow PLA foams to have a higher crystallinity than neat PLA, and consequently a higher temperature resistance [40]. Thermal annealing is also possible in some cases. In this line, an increase in the compression strength of PET foam of up to 130% at 100 °C has been obtained after an overnight temperature soak at that temperature [113], which extends the service temperature of the foam past its T_g_.

*In summary, all non-toxic and recyclable polymers and gases would meet the C2C criteria. The need to replace widely used foams based on hazardous or non-recyclable raw materials, or the integration of foams in multifunctional sandwich structures, triggers the need for foaming alternative polymers. Characterization data on (i) polymer melt–gas systems, including extensional rheology, solubility and diffusivity of the gas in the melt, superficial tension, plasticization effects, and crystallization kinetics, are required to assess foamability and optimize foaming, and (ii) ageing of foams, to assess their service life and benchmark foams according to their entire lifecycle, are not always available for polymers and polymer melt–gas systems which have not yet been foamed. Research on material characterization is needed to broaden the choice of foamable polymers.*

### 4.2. Optimization of Polymer Foamability

The previous section discussed that the foamability of many polymers remains to be explored. Nevertheless, it is recognizable that the foaming of the most easily-foamed polymers has been achieved. However, how can properties equivalent to state-of-the-art polymeric foams be achieved, based on materials that are already ‘second choice’ in their foaming abilities? In this section, strategies to overcome the most commonly reported challenges are revised.

#### 4.2.1. Overcoming Poor Melt Strength

As compiled in the previous section, the lack of suitable melt rheology is a common challenge. Melt rheology can generally be modified by additives [64,114], changes in molecular weight and molecular weight distribution [64,90] and cross-linking, either chemically [115,116,117,118] or by electron beam irradiation [119,120,121]. Extensive cross-linking turns the matrix into a thermoset, conditioning its recyclability [115]. Again, additive minimization is desired to facilitate broad recyclability. Therefore, the focus of this review is placed on modification of the polymer and processing:

- Reactive extrusion: branching and chain extending.

Branching polymers have been reported to have better foaming properties, as branches contribute to melt strain hardening as they prevent the macromolecules to disentangle at the same rate as exponential deformation during the bubble growth phase [74,94,122]. Therefore, resin rheology is reported to be improved through reactive branching or chain extension, resulting in successful foaming [58,65,122,123], adding branching agents or chain extenders during the extrusion process [54,122,124]. It may be argued that adding branching and chain extending agents is additivation, and modification of the matrix would complicate the later polymer sorting and recycling. There is a thin line between additives and reactants that could lead to a definitions debate. However, C2C does strive for tailor-made design in contraposition to “one-size-fits-all” through its Celebrate Diversity tenate [4] (see Section 2.2). At the same time, it should be highlighted that, even in thermoplastics, successive washing and reprocessing cycles lead to polymer degradation and chain scissoring, as well documentation for the case of PET [125,126]. Endless recycling in a fully closed loop cannot be achieved in practical terms. Therefore, reactive branching and chain extending can also be used to improve the rheology and allow the use of recycled resins.

However, care must be taken in the design and material selection for the branching process. As reported in [63], relatively high concentrations of the unreacted branching agent often remain in the final product, which may restrict certain uses or compromise its long-term stability. A certain level of cross linking may occur by using chain extenders [127], and its impact towards successive recycling loops needs to be assessed. Toxic chemicals like diisocyanates have been explored as chain extenders for PLA [128,129,130,131], overturning environmental benefit claims on the developed material, stemming from its bio-based and bio-degradable nature. Pyromellitic dianhydride, which has been found to successfully improve the rheology of PET and recycled PET as a chain extender [132,133,134], has recently been reported to be suspected of causing occupational asthma [135].

Chain extending and branching through reactive extrusion have been found a successful technique to improve the melt strength and enable successful foaming of polymers. Research and development efforts are required to identify and/or develop nontoxic chain extenders and branching agents:

- Improving foamability properties during the resin manufacturing phase.

**Figure 6:**
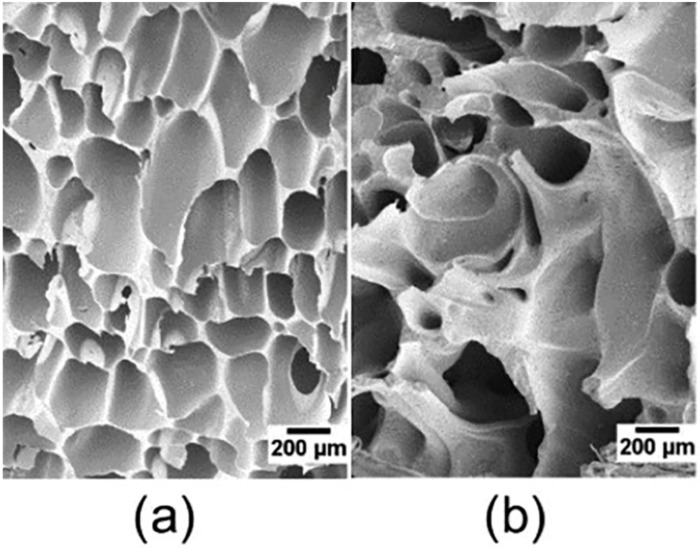
Foams produced with long-chain branched PP (**a**) and linear PP (**b**) under the same foaming conditions, reproduced from [89] with permission from Wiley.

With the knowledge on resin properties required for successful foaming, optimization could be conducted during the resin manufacturing phase, which will later reduce the need for additives. The development of high melt strength polypropylene (HMS-PP) has been an area of great industrial research activity in recent years and has enabled its foaming [32,89,136,137]. The better foam morphology obtained with HMS branched PP vs. linear PP can be visualized in Figure 6. Linear HMS-PP has been developed for foaming [138] but most HMS-PP arise from the addition of long branches, produced through gamma irradiation [139], Ziegler-Natta [140] or metallocene catalysis [141,142]. Metallocene catalysts [56] are promising to tailor the resin structure to fit foaming processes, as they allow better control on the monomer and molecular weight distribution [143,144]. Metallocene catalysts not only can allow for custom design and engineering of new polymeric materials but also offer environmental benefits as they allow for producing polyolefins with milder process conditions, do not contain toxic heavy metals, and the additional chemicals and compounds used for their heterogenization are chemically inert [144].

The development of HMS-PP was demand driven, given the interest of foaming PP. Cited properties that justify this interest include higher rigidity compared to other polyolefins [89,136], higher strength than PE [32,136], superior impact strength compared to PS [32,136], higher service temperature range and good temperature stability [89] compared to PE and PS [32,136], and competitive material cost [32]. Interest in foaming other polymers may grow, sparked by the need to replace a material for environmental or regulatory aspects, as in the case of PU, or as a result of broader availability of properties data (Section 4.1). As the uptake of circular product design increases, another driver would be to match the foam’s polymer with other material layers within sandwich structures [62].

*It is expected that demand will drive the tailoring of resin properties as they become the focus of foaming research:*

- Optimizing the processing tools.

**Figure 7:**
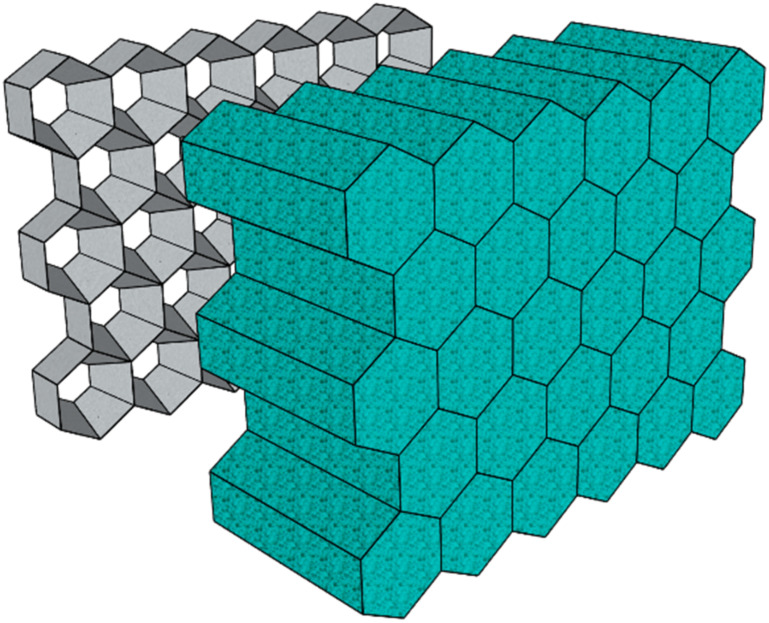
Collided foam strands exiting a multi-orifice die.

Another strategy to overcome the low melt strength is the optimization of the processing tools. A success story is strand foam extrusion technology [139]. A multi-orifice die that produces several individual foam strands is used. These are then pressed together to yield low-density foam sheets, as represented in Figure 7. This tooling facilitates expanding and stabilizing low-melt strength polymers, allowed the development and commercialization of PP foam plank products [137] and is currently the state-of-the-art technology for commercial PET foam [145].

Optimization of processing tools is an effective strategy to advance polymer foaming. Significant research efforts are needed in this area, for example for the manufacturing of nanocellular foams.

#### 4.2.2. Processing Strategies towards Cell Stabilization

Having addressed the melt strength optimization to support the bubble growth, the next stage for allowing successful foaming would be to support and optimize the cell stabilization step and dimensional stability of the obtained foam. CO_2_ is a suitable blowing agent given its high solubility and produced plasticization, which allows to foam at lower temperatures and supports the stabilization step when the plasticization is lost upon desorption [45]. However, the shrinkage and collapse of the cellular structure of the resulting foams due to its high diffusivity in the hot polymer matrix are problematic, in particular with low rigidity [71,72] and elastomeric [76,77] polymers. A long-established strategy to overcome this has been the use of long chain hydrocarbons as blowing agents [57,136]. Long chain blowing agents have low volatility resulting in low diffusivity [138], while CO_2_ has a higher diffusivity related to its smaller molecular size [146]. This slower gas diffusion contributes to a better control of the cell stabilization step. The flammable nature of these blowing agents [71,72] and the consecutive need for flame retardants trigger the need to move towards inert gases, as discussed in Section 4.1. Another common procedure is the addition of surfactants and permeability modifiers [56,57], examples of which are stearic acid amide, glycol monostearate, and glycine fatty acids [56]. Given the criteria to avoid the use of additives, a processing strategy to prevent or minimize gas escape is to reduce the temperature, thereby reducing the diffusivity [147]. Freezing extrudate skin by lowering the die temperature has been reported as a successful strategy to prevent gas escape and stabilize cell structure [136,148]. In a batch foaming study [33], reduction of saturation pressure is suggested as a strategy to prevent cell collapse due to gas escape. However, this comes together with a lower pressure drop rate, which has been identified as a parameter that correlates with cell density [149]; therefore, a compromise between the two needs to be found.

A study on the effect of confined foaming of rubbery elastomers in a mold vs. free foaming with CO_2_ has recently been published [76]. It concluded that, through confined foaming with permeable molds, dimensionally stable foams could be obtained with a higher cell uniformity but also density than in the case of free foaming. With impermeable molds, dimensionally stable foams with higher expansions could be achieved due to the restriction of the gas escape. However, post expansion and relaxation could occur after removal from the mold in some cases.

CO_2_-N_2_ mixtures have been reported to be used to tailor and optimize the resulting foam microstructure [33] and avoid post-foaming shrinkage while still using inert gases only [72,77], as a synergetic effect is achieved between cell nucleation enhanced by CO_2_ solubility and minimized shrinkage due to lower diffusivity of N_2_.

**Figure 8:**
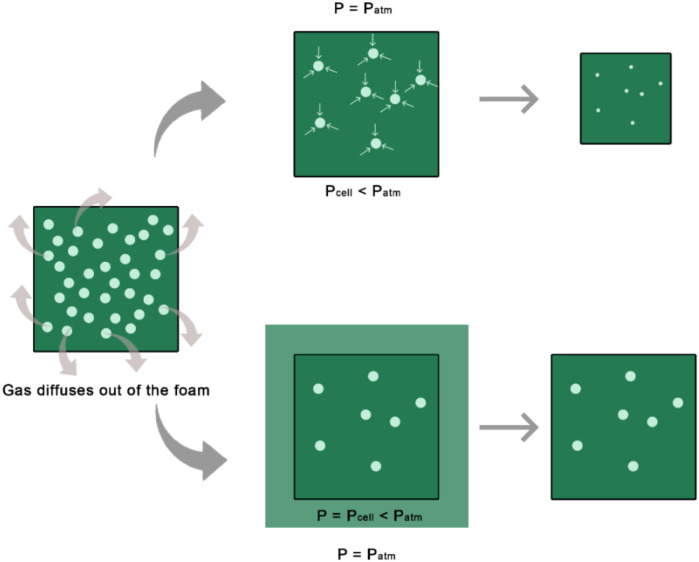
Negative pressure created within the cells as the gas diffused from the hot skin of the foam (top scheme). Lowering ambient pressure during the post-foaming ageing phase could counterbalance the pressure decrease inside the cells and prevent shrinkage.

A novel proposed anti-shrinkage strategy is to reduce ambient pressure during the post-foaming ageing phase [77] to counterbalance the decrease in pressure inside foam cells due to CO_2_ escape, as schematically represented in Figure 8. This is an excellent example of how creative use of physical and processing parameters can be used to avoid the use of additives or hazardous raw materials. The work [77] also modelled the effect of cell shape on the shrinkage, concluding that circular cells present better dimensional stability, followed by hexagonal and last square shaped cells. This would also be an interesting strategy, although the obtention of particular cell shapes in practical terms remains a challenge.

*Identified strategies to control the dimensional stability of cellular structures compatible with CE include optimizing the foaming pressure and temperature, reducing ambient pressure during post-foaming, and the use of molds. The impact of these strategies on the obtained foam morphology and skin formation and optimization and potential trade-offs between the different parameters in play need to be assessed and adjusted for each practical case.*

### 4.3. Tailoring Properties through Foam Processing and Cellular Structure

The cellular structure has a decisive influence on the thermal and mechanical properties of foams [53,108]. The foam morphology is characterized by cell size, shape and population density, cell size variability, existence and morphology of skin and foam bulk density, all of which are function of the selected polymer–gas system, the processing parameters and the processing tools.

As for thermal properties, the different contributors to the foam’s effective thermal conductivity were presented in Equation (1). After considering the physical properties of the foam’s raw materials, the thermal conductivity can be modified through the cell morphology as it affects the thermal radiation. It is influenced by both the cell size and cell shape.

Concerning the shape, increasing the cell isotropy is reported to reduce thermal conductivity, related to effects of polymer chain alignments in anisotropic foams [150]. Differently, a reduction of the thermal conductivity from 0.035 W/mK to 0.025 W/mK was achieved through the production of very elongated cells, with an aspect ratio ideally larger than 2 [151]. This highly anisotropic cellular structure was achieved through the choice of a multi-hole die geometry and high pulling speed during the extrusion process, together with a high melt strength resin [151].

The reduction of the cell size contributes to favorable insulating properties of the foam in different ways. On one hand, small cell size slows the diffusion of the blowing agent out of the foam [33], reducing the thermal aging of the foam with time. On the other hand, reducing the cell size has been experimentally proven as an effective way to reduce thermal conductivity of polymeric foams [150,152] irrespective of the blowing agent [153], and related to the radiative heat transfer within the foam [154]. This is explained by the higher number of reflections occurring in foams with smaller cell size [53]. This however no longer holds for highly expanded nanocellular foams, as the thin cell walls and struts may become highly transparent to thermal radiation, not being able to attenuate it [155,156]. An optimum between volume expansion and cell size needs to be reached to minimize thermal conductivity in cellular nanocellular foams [155]. A quantitative step forwards to reducing thermal conductivity would be by using the Knudsen effect, which would be achieved when the gas molecules do not interact with each other but collide with the molecules of the surrounding solid, reducing the energy transfer through the gas molecules [157]. That is, when cell size is comparable or smaller than the mean free path of the gas [107,158]. This has been demonstrated to occur in nanocellular polymeric foams, with cell diameter <700 nm [159]. While research on the foaming of nanocellular polymers is ongoing [159,160], so is the understanding of their structure–properties relationships. The effect of the cell size distribution on the thermal conductivity of nanocellular foams has been recently modelled [161], highlighting the interest on bimodal structures. This is because, while nanometric cells reduce the conductivity through the gas phase, microcellular pores reduce the conduction through the solid phase.

**Figure 9:**
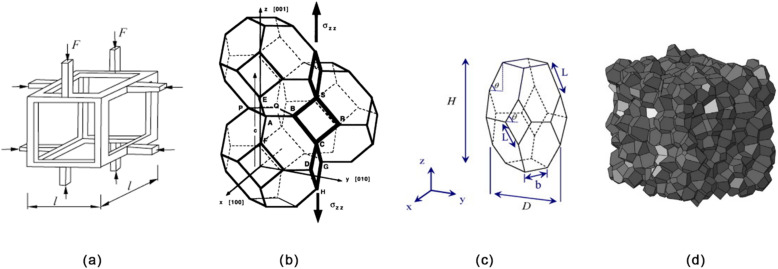
(**a**) Cube cell model, reproduced from [162] with permission; (**b**) Kelvin cell model, reproduced from [163] with permission; (**c**) Kelvin cell model, which accounts for cell anisotropy, reproduced from [165] with permission; (**d**) Laguerre tessellation model of a foam, reproduced from [167] with permission.

The cellular structure not only affects the thermal properties but also the mechanical ones, which is key in many multifunctional applications of cellular plastics. Early work by Gibson and Ashby [162] modeled the cell as a cube and related the Youngs modulus (E) to the relative density of the foam and the E of the cell wall material. Modeling the mechanical behavior of foams has been progressively refined to adjust the cell shape to that of a Kelvin cell [163], account for cell anisotropy [164,165], account for cell size in addition to relative density [166] and, with the use of Laguerre tessellation models, variation of cell size and cell wall thickness [167]. These models are represented in Figure 9.

These models provide the indication that E scales with relative density [53], decreases with increasing cell size and variability of cell wall thickness [167], and can be maintained constant while reducing density if cell size is reduced [166]. In this line, a breakthrough in the mechanical properties of polymeric foams came with the development of microcellular foams pioneered at MIT during the 1980s [168,169,170]. They refer to those with cell size in the order of 10 µm and a cell population density of the order of 10^8^ cells/cm^3^. Their development was aimed at reducing the amount of polymer used in mass produced products, as well as their transportation costs. The impact strength of the foams was found to be increased by 6 to 7 times versus that of solid polymers of the same linear dimensions, related to improved craze initiation and crack blunting [171], and appeared to have higher specific strength and improved fatigue life [170]. It appears clear that the reduction of the cell size results in improved mechanical and insulating performance of polymeric foams, and should be a design target. The nucleation step is key for the achievement of large numbers of small cells and as such has been the focus of research since early stages of microcellular foaming. According to classical nucleation theory, two mechanisms are possible for a second phase nucleation in a primary phase: homogeneous nucleation, which would occur when a second component reaches a critical amount, forming a stable second phase, or heterogeneous nucleation, when a third phase is formed in the interphase of two other phases [172], such as an additive in a polymer matrix. The activation energy for heterogeneous nucleation is lower, resulting in the earlier nucleation of heterogeneously nucleated bubbles than of homogeneously nucleated bubbles [173]. The number of nucleated bubbles is a function of the concentration of heterogeneous nucleation sites and their relative effect on the nucleation activation energy [173]. Additives can enhance nucleation by providing the presence of an interface, but also by reducing the surface tension of the polymer [174]. With additive minimization in the focus of this article, homogeneous nucleation can be enhanced by increasing the pressure drop, the driving force for bubble nucleation, as can be recognized from the following general expression for nucleation rate (*N*) [33]:

(2) $$N=M\cdot B\;\exp\left[-\frac{16\pi \sigma^{3}}{3k_{B}T{\left(P_{v}-P_{L}\right)}^{2}}\right]$$

where *M* and *B* are increasing functions of the gas concentration and of the gas diffusivity, respectively. *k_B_* is the Boltzmann constant, and *P_v_* and *P_L_* are the equilibrium gas pressure and the pressure in the liquid phase.

**Figure 10:**
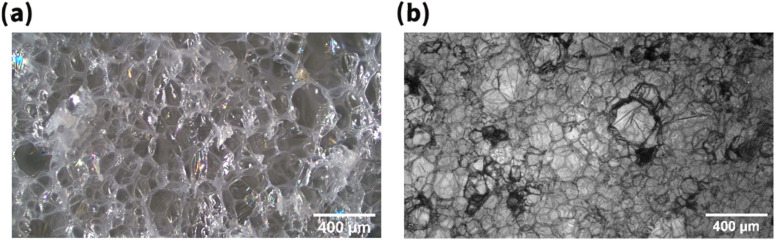
PB-1 foam obtained with a P drop of 49 bar (**a**) and of 99 bar (**b**).

This effect is clearly observed in [175]. By modifying the foaming pressure drop, polybutene-1 foams with similar density were produced, but with very different microstructure: cell size reduction by a factor of 1/6 and cell population density increase by a factor 66 were achieved by increasing the foaming pressure from 50 to 100 bars. This is illustrated by the two micrographs in Figure 10.

A further parameter that affects the thermodynamic instability driving bubble nucleation is the pressure drop rate, as first proposed by Park et al. [149]. It affects the competition between cell nucleation and growth because the gas that contributes to cell growth is minimized by maximizing the pressure drop rate. The fundamental role of the pressure drop rate in the density of polymeric foam cell population has been confirmed by later authors [176], and efforts have been made to explore and understand the boundary conditions under which the number of nucleated cells can increase with increasing pressure drop rate. Using a miniature batch foaming apparatus that achieves pressure drop rates of up to 500 MPa/s [177], Tammaro et al. [178] report that the density of the cell population increases linearly with the pressure drop rate on a bi-logarithmic scale. Studies on the effect of maximizing the pressure drop rate have typically been carried out in batch apparatus [176,177,179], and R&D efforts are needed to expand the technology for commercial foam manufacturing under these process conditions.

**Figure 11:**
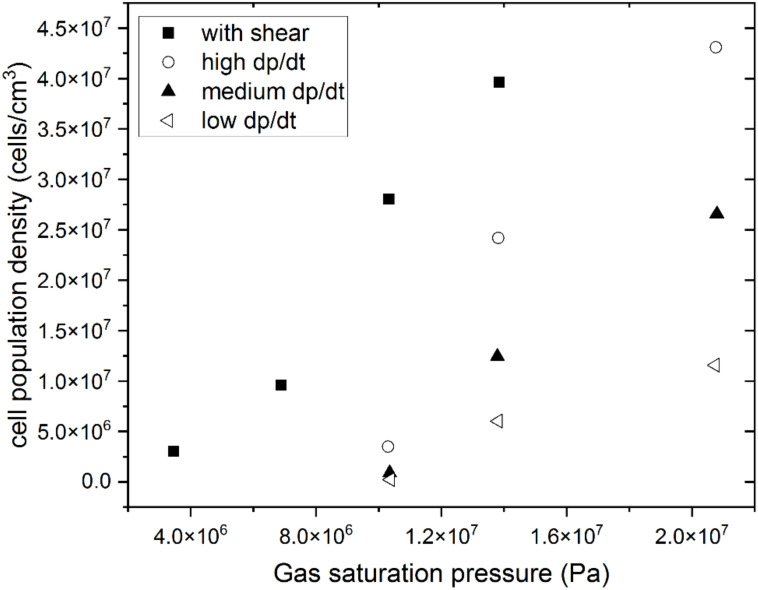
Cell population density as a function of gas saturation for HDPE foamed with different pressure drop rates and the application of shear [180].

A further parameter that plays a role in cell nucleation is the effect of shear forces in the melt, which occur at the die in an extrusion process. These forces have been reported to promote nucleation and are interpreted through a cavity model, where the presence of high shear would “pull” the gas phase out of the solid cavity [51]. They are found to have a greater impact on cell nucleation than the pressure drop rate, with particular relevance at low saturation pressures, when the pressure drop is an insufficient driving force [51,180]. This can be observed in Figure 11.

Although more recent studies generally confirm these observations [181], in this review of the literature, no mechanistic or phenomenological model has been found relating shear to cell nucleation.

A more comprehensive understanding of the mechanism is needed to fully use this parameter in morphology optimization. Acting shear forces are related to the polymer melt–gas solution rheology, highlighting the need for characterization data as presented in Section 4.1.

Mechanical and thermal properties of polymeric foams are generally improved with the reduction of cell size, and can be tuned through cell size variability. Pressure drop rate and acting shear have been confirmed as processing parameters that contribute to the obtention of microcells, by promoting cell nucleation. Research is needed for a more detailed understanding of the effects of shear on cell nucleation. R&D efforts are needed to scale up manufacturing tools to obtain high pressure drop rates in commercial scale manufacturing.

## 5. Conclusions

For the development of polymeric foams consistent with circular product design, the following strategies and research needs have been identified during this review:

- To broaden the selection of polymeric foams, research on material characterization is needed, on (i) polymer melt–gas systems, including extensional rheology, solubility and diffusivity of the gas in the melt, superficial tension, plasticization effects and crystallization kinetics to assess foamability and optimize foaming, and (ii) ageing of foams, to assess their service life and benchmark foams according to their entire lifecycle;
- Chain extending and branching through reactive extrusion has been found a successful technique to improve the melt strength and enable successful foaming of polymers. Research and development efforts are required to identify and/or develop nontoxic chain extenders and branching agents;
- Polymer chain configuration could be tailored for foaming applications during the manufacturing phase. It is expected that the demand will drive the tailoring of resin properties of alternative polymers as they become the focus of foaming research;
- Optimizing the processing tools is an effective strategy to advance on polymer foaming. The breaker plate could be used for the foaming of low melt strength polymers. Significant research efforts are required in this area, to allow the commercial manufacturing of, i.e., nanocellular foams;
- Identified strategies to control the dimensional stability of cellular structures include the foaming pressure and temperature and the use of molds. The impact of these strategies on the obtained foam morphology and skin formation, and the optimization and potential trade-offs between the different parameters in play need to be assessed and adjusted for each practical case;
- Mechanical and thermal properties of polymeric foams are generally improved with the reduction of cell size and can be fine-tuned through cell size variability. The pressure drop rate and acting shear have been confirmed as processing parameters that contribute to the obtention of microcells by promoting cell nucleation. Research is needed to gain a more detailed understanding of the effects of shear on cell nucleation. R&D efforts are needed to scale up manufacturing tools to obtain high pressure drop rates.

## Data Availability

Data available through the reported references.
